# Neural Field Theory of Corticothalamic Prediction With Control Systems Analysis

**DOI:** 10.3389/fnhum.2018.00334

**Published:** 2018-09-10

**Authors:** Tahereh Babaie Janvier, Peter A. Robinson

**Affiliations:** ^1^School of Physics, University of Sydney, Sydney, NSW, Australia; ^2^Center of Excellence for Integrative Brain Function, University of Sydney, Sydney, NSW, Australia

**Keywords:** brain dynamics, cortex, thalamus, neuronal prediction, neural field theory, neuro-dynamic, control systems, filter

## Abstract

Neural field theory is used to model and analyze realistic corticothalamic responses to simple visual stimuli. This yields system transfer functions that embody key features in common with those of engineering control systems, which enables interpretation of brain dynamics in terms of data filters. In particular, these features assist in finding internal signals that represent input stimuli and their changes, which are exactly the types of quantities used in control systems to enable prediction of future input signals, and adjustment of gains which is argued to be the analog of attention in control theory. Corticothalamic dynamics are shown to be analogous to the classical proportional-integral-derivative (PID) filters that are widely used in engineering.

## 1. Introduction

The brain must carry out functions that implement attention to external stimuli, prediction of their future course, and decision with regard to actions to take in response, including interventions to control aspects of the environment. In the natural environment, a large fraction of visual processing deals with the prediction of spatiotemporal trajectories of objects and the taking of actions to either intercept or avoid them. For example, an organism may seek to capture and eat an object in its environment or to avoid being captured and eaten. These decisions thus differ markedly from the binary decision and discrete classification tasks widely studied in cognitive neuroscience.

With regard to attention to multiple sensory streams, there is extensive evidence that attention paid to individual streams is apportioned on an approximately Bayesian basis (Feldman and Friston, 2010; Friston, 2010). Notably, the weight placed on a particular stream is approximately inversely proportional to its variance (Feldman and Friston, 2010; Friston, 2010). These biological observations, along with advances in machine learning and neuroscience, have motivated a plethora of models of attention and prediction in the brain, each with Bayesian features, with most motivated by cortical neuroanatomy and neurophysiology. However, none of the proposed frameworks has yet been shown to be fully implementable in the brain's tissues.

One example of this point is the model of Rao and Ballard (1999), who used the Kalman filter to model visual information processing in the brain. A Kalman filter is a Bayesian approach that applies when all the distributions of internal states, external data, and their uncertainties, are Gaussian distributed and the underlying internal dynamics are linear. Rao and Ballard showed that a Kalman filter could accomplish some prediction outcomes, and demonstrated neuronal implementation of some of its steps, but did not explain how its all complex matrix operations would actually be implemented in the brain.

Helmholtz (1867) suggested that the brain predicts its inputs and adjusts an internal model to minimize mismatches between these predictions and external subsequent inputs. Friston and coworkers elaborated on this idea to develop Bayesian estimation schemes with both bottom-up and top-down signaling, and proposed that the brain employs a hierarchical Bayesian approach for perception and, crucially, action (Lee and Mumford, 2003; Friston, 2005, 2010; Hawkins and Blakeslee, 2007; Daunizeau et al., 2010b,a). The common theme of these approaches rests upon the minimization of the mismatch between top-down predictions and bottom-up expectations. Subsequent work Mathys et al. (2011, 2014) expanded classic Bayesian inference to optimize the precision of prediction errors which enabled them to include reinforcement learning into the ensuing *hierarchical Gaussian filter* (HGF) for perception, attention, and action. This family of models has been shown to be able to carry out a number of sophisticated prediction tasks, but this raises a number of questions. Specifically, they rely on the brain being able to determine, store, and update multivariate probability distributions of Bayesian priors, to carry out multidimensional integrations over these distributions, and to compute large scale matrix operations, all in real time. Although these problems have been recognized, and suggestions have been made to simplify the evaluations by means of reduced moment-based representations, for example (Bastos et al., 2012; Pouget et al., 2013; Mathys et al., 2014; Friston et al., 2017), it has yet to be established that the brain can carry out the necessary calculations.

The current state of affairs is thus that each proposal in the literature is motivated by the real brain, but relies in places on mathematical steps which have no established implementation in neural tissue. Hence, although these schemes have many plausible features, and many interesting applications have been demonstrated, none has been shown to be fully realizable in the brain.

Motivated by the need for a formulation of brain dynamics that is physiologically realizable, analytically and numerically tractable, and experimentally testable, we take a different approach. Instead of deciding on a favored mathematical formulation and assuming that it works in the brain, the present work takes the physically motivated reverse approach of first analyzing realistic corticothalamic responses to simple visual stimuli using neural field theory (NFT). This enables us determine what filter properties they exhibit, rather than advancing a predetermined model. We thus analyze the corticothalamic system, focusing on the response of primary visual cortex V1 to spatially unstructured stimuli which has been widely used in visual flicker experiments to probe steady state visual evoked potentials (SSVEPs) (Spekreijse, 1966; Spekreijse et al., 1973; Herrmann, 2001; VanRullen and Macdonald, 2012). In doing this we postpone application to more complex stimuli in order to focus on establishing the first example of a fully neurally implementable scheme for prediction, followed in a subsequent paper by attention and decision. This involves treating the brain first and foremost as a physical system that is responding to its environment, rather than as a computer or abstract information processing engine. Some of the dynamical processes that we uncover may be able to be fruitfully interpreted as information processing or computation, but such interpretations are subject to the fact that the brain is a physical object.

We first employ NFT to model the corticothalamic system and determine the extent to which its dynamics can be interpreted within a control systems framework which has the potential to encompass prediction, gain tuning, and control. Most control systems and Bayesian schemes such as Kalman filtering are reduced or constraint form of Bayesian learning under optimal estimation theory (Chen, 2003). In carrying out our analysis, we determine which signals within the system represent input stimuli and their rates of change, because these are the classes of quantities used in control systems to enable prediction of future input signals, and adjustment of gains which is argued to be the analog of attention in control theory. The finding of analogous quantities in the corticothalamic system enables interpretation of its dynamics in terms of control systems, and assists in localizing the structures in which gain control is possible in principle. In a forthcoming paper, we will use these findings to propose physiologically-based mechanisms for attention and control via feedbacks. At each stage, we are explicit about the neural implementation of the mechanisms.

This paper is structured as follows. Section 2 briefly outlines the corticothalamic model of the brain using the neural field theory and then we obtain transfer functions for the corticothalamic model. The transfer functions are analyzed and data filter interpretations of them are presented at Section 3. Section 4 concludes and discusses future work.

## 2. Materials and methods

### 2.1. Corticothalamic neural field theory

In this section we outline the essentials of NFT, then apply it to a generalized corticothalamic model. For more details see Robinson et al. (2002, 2004).

#### 2.1.1. Neural field theory

NFT averages over short spatial and temporal scales to obtain equations for the evolution of dynamical variables within each neural population *a*, which are the local mean cell-body potentials *V*_*a*_, the mean rate of firing at the cell body *Q*_*a*_, and the propagating axonal pulse rate fields ϕ_*a*_.

The mean firing rates *Q*_*a*_ are related to the cell body potentials *V*_*a*_(**r**, *t*), relative to resting, by

(1) $$\begin{array}{l} Q_{a}\left(\text{r},t\right)=S\left[V_{a}\left(\text{r},t\right)\right], \end{array}$$

where *S* is a sigmoid function that increases smoothly from 0 to *Q*_max_ as *V*_*a*_(**r**, *t*) increases from −∞ to ∞. An approximation of this function is (Wilson and Cowan, 1973; Freeman, 1975)

(2) $$\begin{array}{l} S\left[V_{a}\left(\text{r},t\right)\right]=\frac{Q_{\text{max}}}{1+\exp\left\{-\left[V_{a}\left(\text{r},t\right)-\theta \right]/\sigma^{\prime }\right\}}, \end{array}$$

where θ is the mean neural firing threshold and $\sigma^{\prime }\pi /\sqrt{3}$ is the standard deviation of the difference between the steady state depolarization of individual neurons and their thresholds. Effectively, the threshold response of a single neuron is smeared out to yield a sigmoid when averaged over the population.

Signals arriving at neurons of type *a* stimulate neurotransmitter release at synapses. This is followed by propagation of voltage changes along dendrites and soma charging, with dynamics that spread the temporal profile of the signals. The total cell body potential can thus be written

(3) $$\begin{array}{l} V_{a}\left(\text{r},t\right)=\underset{b}{\sum }V_{ab}\left(\text{r},t\right), \end{array}$$

where the subscripts on *V*_*ab*_ distinguish the different combinations of afferent neural type and synaptic receptor, and

(4) $$\begin{array}{l} D_{ab}\left(t\right)V_{ab}\left(\text{r},t\right)=\underset{b}{\sum }N_{ab}s_{ab}\phi_{b}\left(\text{r},t-\tau_{ab}\right), \end{array}$$

where the differential operator *D*_*ab*_ that governs the temporal response of *V*_*ab*_ to afferent pulse rate fields ϕ_*b*_ is

(5) $$\begin{array}{l} D_{ab}\left(t\right)=\frac{1}{\alpha_{ab}\beta_{ab}}\frac{d^{2}}{dt^{2}}+\left(\frac{1}{\alpha_{ab}}+\frac{1}{\beta_{ab}}\right)\frac{d}{dt}+1. \end{array}$$

The operator *D*_*ab*_ encapsulates the rates β_*ab*_ and α_*ab*_ of the rise and fall, respectively, of the response at the cell body. On the right of Equation (4), *N*_*ab*_ is the mean number of synapses on neurons a from neurons of type *b*, *s*_*ab*_ is the mean time-integrated strength of soma response per incoming spike, and ϕ_*b*_(**r**, *t*−τ_*ab*_) is the mean spike arrival rate from neurons *b*, allowing for a time delay τ_*ab*_ due to anatomic separations between discrete structures. The overall connection strength between two neural populations is ν_*ab*_ = *N*_*ab*_*s*_*ab*_.

Each part of the corticothalamic system gives rise to neural pulses, whose values averaged over short scales form a field ϕ_*a*_(**r**, *t*) in our model that propagates at a velocity *v*_*a*_. To a good approximation, ϕ_*a*_(**r**, *t*) obeys a damped wave equation whose source of pulses is *Q*_*a*_(**r**, *t*) (Jirsa and Haken, 1996; Robinson et al., 1997), with

(6) $$\begin{array}{l} D_{a}\left(\text{r},t\right)\phi_{a}\left(\text{r},t\right)=Q_{a}\left(\text{r},t\right), \end{array}$$

where the spatiotemporal differential operator *D*_*a*_(**r**, *t*) is

(7) $$\begin{array}{l} D_{a}\left(\text{r},t\right)=\frac{1}{\gamma_{a}^{2}}\frac{\partial^{2}}{\partial t^{2}}+\frac{2}{\gamma_{a}}\frac{\partial }{\partial t}+1-r_{a}^{2}\nabla^{2}. \end{array}$$

Here the damping rate γ_*ab*_ satisfies γ_*a*_ = *v*_*a*_/*r*_*a*_, where *r*_*a*_ and *v*_*a*_ are the characteristic range and conduction velocity of axons of type *a*.

#### 2.1.2. Corticothalamic model

**Figure 1:**
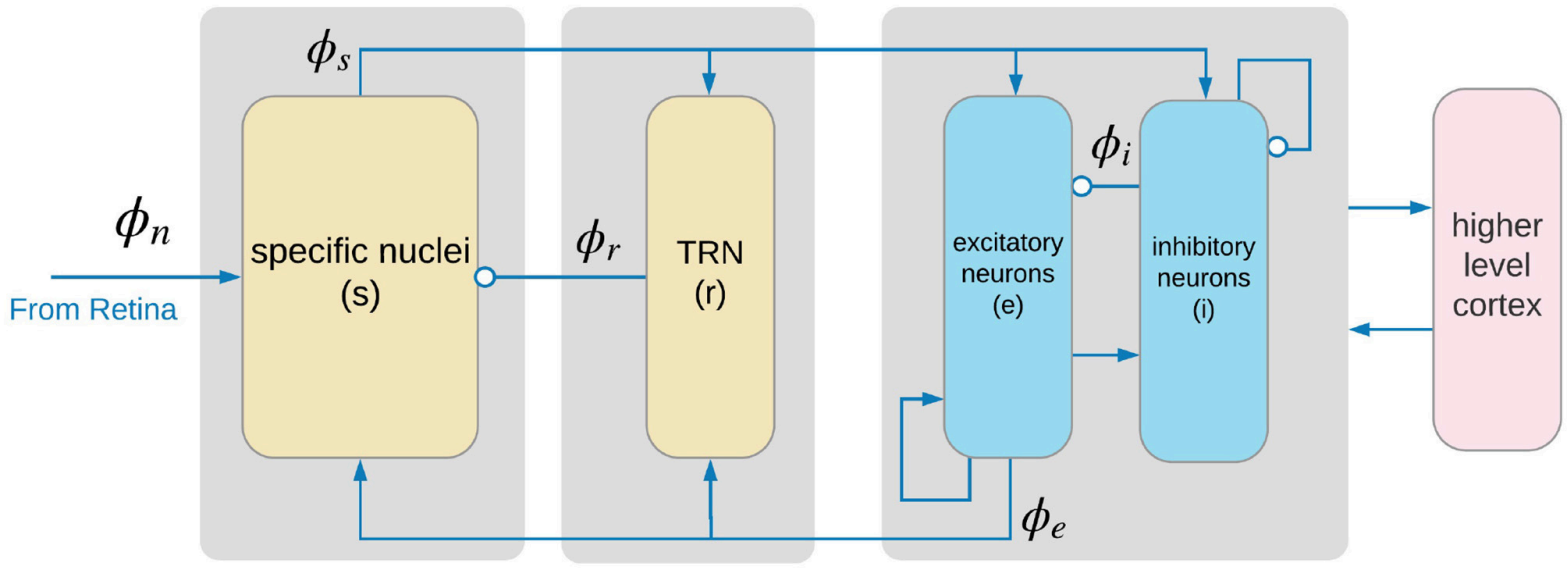
Physiologically based corticothalamic model in which the arrows represent excitatory effects and the circles depict inhibitory ones. The populations are cortical excitatory (*e*) and inhibitory (*i*) neurons, the thalamic reticular nucleus (*r*), thalamic relay neurons (*s*) that project to the cortex, and non-corticothalamic neurons responsible for external inputs (*n*). Gray boxes depict the lateral geniculate nucleus (LGN) (left), the thalamic reticular nucleus (TRN) (middle), and primary visual cortex (right).

Corticothalamic NFT incorporates key anatomic connectivities as shown in Figure 1. The neural populations included are cortical excitatory (*e*) and inhibitory (*i*) neurons, the thalamic reticular nucleus (*r*), thalamic relay neurons (*s*) that project to the cortex, thalamic interneurons (*j*), and noncorticothalamic neurons responsible for external inputs (*n*). In the present case, external inputs are visual, the relevant relay nucleus is the lateral geniculate nucleus (LGN), and its projections are to the primary visual cortex (V1).

The only nonzero values of τ_*ab*_ in our model are the forward delays τ_*es*_ = τ_*is*_ ≈ 20 ms and the backward delays including τ_*se*_ = τ_*je*_ = τ_*re*_ ≈ 60 ms, which correspond to thalamocortical and corticothalamic propagation times, respectively. The use of a single form of *D*_*ab*_ corresponds to the approximation that the mean dendritic dynamics can be described by a single pair of time constants.

In our model, only the axons of excitatory cortical neurons *a* = *e* are long enough to yield significant propagation effects in Equation (7); in the other cases, *r*_*a*_ is so small that the solution of equations above can be approximated by ϕ_*a*_(**r**, *t*) = *S*[*V*_*a*_(**r**, *t*)] = *Q*_*a*_(**r**, *t*). In the cortex, the number of synapses is closely proportional to the numbers of source and target neurons (Robinson et al., 1997, 1998; Braitenberg and Schüz, 1998), which implies that ν_*ee*_ = ν_*ie*_, ν_*es*_ = ν_*is*_, and ν_*ei*_ = ν_*ii*_. Table 1 lists the nominal values estimated for the corticothalamic model parameters; the details can be found in Robinson et al. (2004).

**Table 1:** Physiologically estimated model parameters for normal adults in the alert, eyes-open state (Robinson et al., 2004).

**Quantity**	**Description**	**Value**	**Unit**
*Q*_max_	Max firing rate	250	s^−1^
θ	Firing threshold	15	mV
σ′	Threshold spread	3.3	mV
γ_*e*_	Cortical damping rate	100	s^−1^
α_*ab*_	Inverse decay time	80	s^−1^
β_*ab*_	Inverse rise time	320	s^−1^
τ_*es*_	Forward delay time	20	ms
τ_*se*_	Feedback delay time	60	ms

### 2.2. Steady states, linearity, and transfer functions

The NFT equations are nonlinear in general, and highly nonlinear phenomena like epileptic seizures have been studied with them (Breakspear et al., 2006). Normal brain states have been shown to correspond to spatially uniform steady states of corticothalamic NFT, and are obtained by setting all time and space derivatives to zero (Robinson et al., 2002, 2004; Abeysuriya et al., 2015). Stable steady state solutions are interpreted as representing the baseline of normal activity, which yields firing rates in accord with experiment (Robinson et al., 2002, 2004). Time dependent brain activity is then represented by linear perturbations from the steady states—an approximation that has reproduced a host of experimental phenomena, including evoked responses (Robinson et al., 1997, 1998, 2002, 2004, 2005, 2008; O'Connor and Robinson, 2004; Rowe et al., 2004; Kerr et al., 2008; van Albada et al., 2010; Roberts and Robinson, 2012; Abeysuriya et al., 2015; Sanz-Leon and Robinson, 2017). In this section, we calculate the linear transfer functions of stimuli to corticothalamic populations.

#### 2.2.1. Linear dynamics

The low firing rate of steady-states have been identified with normal states of brain activity (Robinson et al., 1997) and nonlinear terms are only found to be significant in very strong stimulation conditions (Herrmann, 2001; Roberts and Robinson, 2012; Abeysuriya et al., 2015). The criterion for linear approximation to be valid is that voltage perturbations should be significantly less than σ′/3. Linear perturbations relative to uniform steady-state values $\phi_{a}^{\left(0\right)}$, where *a* = *e, i, r, s*, and $V_{a}^{\left(0\right)}$ approximately obey the damped wave equation

(8) $$\begin{array}{l} D_{ab}\left(\text{r},t\right)\phi \left(\text{r},t\right)=\rho_{a}V_{a}\left(\text{r},t\right), \end{array}$$

where we henceforth use the symbols ϕ_*a*_ and *V*_*a*_ to denote the linear perturbations of these quantities relative to their steady-state values, unless otherwise indicated, and ρ_*a*_ = *dS*(*V*_*a*_)/*dV*_*a*_, evaluated at $V_{a}^{0}$. The external field ϕ_*n*_ which drives the brain via the relay nuclei also comprises a steady-state component $\phi_{n}^{\left(0\right)}$ plus a time-varying signal that causes the response, denoted by ϕ_*n*_. The differences from threshold voltage variations of about ±σ′/3 are therefore needed before nonlinear terms become appreciable relative to the linear ones. This yields the variations of order 1*mV*, or slightly larger, which corresponds to approximately a 2-fold firing rate bound. Detailed analysis of the model with respect to these parameters can be found in Robinson et al. (2004).

Operation with *D*_*ab*_ on both sides of Equation (8), plus use of Equation (4), yield

(9) $$\begin{array}{l} D_{ab}\left(t\right)D_{ab}\left(\text{r},t\right)\phi_{a}\left(\text{r},t\right)=\underset{b}{\sum }G_{ab}\phi_{b}\left(\text{r},t-\tau_{ab}\right), \end{array}$$

with

(10) $$\begin{array}{l} G_{ab}=\rho_{a}\nu_{ab}=\rho_{a}N_{ab}s_{ab}. \end{array}$$

The gain *G*_*ab*_ is the response in neurons *a* due to unit input from neurons *b*; i.e., the number of additional pulses out for each additional pulse in.

A transfer function is the ratio of the output of a system to its input in the linear regime. Either the Laplace or Fourier transform can be used to determine transfer functions, but the former is more widely used in engineering control theory, particularly to analyze responses to impulses. To derive transfer functions one may apply the Laplace transform to both sides of Equation (9) to transform it from time *t* to complex frequency *s*. The unilateral Laplace transform is defined by Ogata and Yang (1970)

(11) $$\begin{array}{l} L\left[f\left(t\right)\right]\left(s\right)=f\left(s\right)=\int_{0}^{\infty }f\left(t\right)e^{-st}dt, \end{array}$$

where *s* = −*iω* = Γ−*iΩ* is the complex frequency that parametrizes the response *e*^*st*^. Here, the real quantities Ω and Γ denote the oscillation frequency and growth rate of the response, respectively, so stable responses correspond to *s* lying in the left half of the complex plane, with neutrally stable responses having *s* on the imaginary axis. Alternatively, one may use the continuous Fourier transform F, which is equivalent to evaluating the bilateral Laplace operator with imaginary argument

(12) $$\begin{array}{l} F\left[f\left(t\right)\right]\left(\omega \right)=L\left[f\left(t\right)\right]{|}_{s=-i\omega } \end{array}$$

(13) $$\begin{array}{l} =\int_{-\infty }^{\infty }f\left(t\right)e^{i\omega t}dt. \end{array}$$

Before we calculate system transfer functions, we note that the operator in Equation (4) has the Laplace transform

(14) $$\begin{array}{l} D_{ab}\left(s\right)=\left(1+\frac{s}{\alpha_{ab}}\right)\left(1+\frac{s}{\beta_{ab}}\right). \end{array}$$

We define the corresponding filter function by

(15) $$\begin{array}{l} L_{ab}\left(s\right)=D_{ab}^{-1}\left(s\right)={\left(1+\frac{s}{\alpha_{ab}}\right)}^{-1}{\left(1+\frac{s}{\beta_{ab}}\right)}^{-1}. \end{array}$$

The Laplace transform of Equation (7) is

(16) $$\begin{array}{l} D_{a}\left(\text{k},s\right)={\left(1+\frac{s}{\gamma_{a}}\right)}^{2}+k^{2}r_{a}^{2}, \end{array}$$

where we have also Fourier transformed the spatial Laplacian operator via ∇^2^ → −*k*^2^ where *k* is the wave number.

#### 2.2.2. Transfer function to thalamus from retina

The firing rate (in the spatial-Fourier, temporal-Laplace domain henceforth) in the reticular nucleus is

(17) $$\begin{array}{l} \phi_{r}=G_{re}L_{re}e^{-s\tau_{se}}\phi_{e}+G_{rs}L_{rs}\phi_{s}. \end{array}$$

For relay neurons in the LGN, the firing rate ϕ_*s*_ is

(18) $$\begin{array}{l} \phi_{s}=G_{se}L_{se}e^{-s\tau_{se}}\phi_{e}+G_{sj}L_{sj}\phi_{j}\\ +G_{sr}L_{sr}\phi_{r}+G_{sn}L_{sn}\phi_{n}. \end{array}$$

By substituting ϕ_*r*_ from Equation (17) into Equation (18), the transfer function to thalamus from retina is found to be

(19) $$\begin{array}{l} T_{sn}\left(\text{k},s\right)=\frac{\phi_{s}\left(\text{k},s\right)}{\phi_{n}\left(\text{k},s\right)}, \end{array}$$

(20) $$\begin{array}{l} =\frac{M\left(s\right)U\left(s\right)}{M\left(s\right)R\left(s\right)-N\left(s\right)P\left(s\right)\exp\left[-s\left(\tau_{es}+\tau_{se}\right)\right]}, \end{array}$$

where

(21) $$\begin{array}{l} M\left(s\right)=D_{ee}\left(1-G_{ei}L_{ii}\right)-G_{ee}L_{ee}, \end{array}$$

(22) $$\begin{array}{l} U\left(s\right)=1-G_{srs}L_{srs}, \end{array}$$

(23) $$\begin{array}{l} R\left(s\right)=G_{sn}L_{sn}, \end{array}$$

(24) $$\begin{array}{l} P\left(s\right)=G_{se}L_{se}+G_{sre}L_{sre}, \end{array}$$

(25) $$\begin{array}{l} N\left(s\right)=G_{es}L_{es}, \end{array}$$

where *G*_*abc*_ = *G*_*ab*_*G*_*bc*_, and *L*_*abc*_ = *L*_*ab*_*L*_*bc*_.

#### 2.2.3. Transfer functions to cortex from retina

By setting *a* = *e* and *a* = *i* in Equation (9), the axonal fields of V1 cortical cells are found to obey

(26) $$\begin{array}{l} D_{ee}\phi_{e}=L_{ee}G_{ee}\phi_{e}+L_{ei}G_{ei}\phi_{i}+L_{es}G_{es}e^{-s\tau_{es}}\phi_{s}, \end{array}$$

(27) $$\begin{array}{l} \phi_{i}=L_{ii}G_{ii}\phi_{i}+L_{ie}G_{ie}\phi_{e}+L_{is}G_{is}e^{-s\tau_{es}}\phi_{s}. \end{array}$$

Replacement of ϕ_*i*_ in Equation (26) by means of Equation (27), yields the transfer function to the cortex from the thalamus,

(28) $$\begin{array}{l} T_{es}\left(\text{k},s\right)=\frac{\phi_{e}\left(\text{k},s\right)}{\phi_{s}\left(\text{k},s\right)}=\frac{N\left(s\right)}{M\left(s\right)}\exp\left(-s\tau_{es}\right), \end{array}$$

where *M*(*s*) and *N*(*s*) are as in Equations (21–25). Multiplying Equations (19) and (28) yields the overall transfer function to cortex from retina:

(29) $$\begin{array}{l} T_{en}\left(\text{k},s\right)=\frac{\phi_{e}\left(\text{k},s\right)}{\phi_{n}\left(\text{k},s\right)}, \end{array}$$

(30) $$\begin{array}{l} =T_{es}\left(\text{k},s\right)T_{sn}\left(\text{k},s\right), \end{array}$$

(31) $$\begin{array}{l} =\frac{U\left(s\right)N\left(s\right)\exp\left(-s\tau_{es}\right)}{M\left(s\right)R\left(s\right)-N\left(s\right)P\left(s\right)\exp\left[-s\left(\tau_{es}+\tau_{se}\right)\right]}. \end{array}$$

Replacement of ϕ_*s*_ and ϕ_*e*_ in Equation (27) by means of Equations (19) and (31) yields the transfer function to the inhibitory population from the retina,

(32) $$\begin{array}{l} T_{in}\left(\text{k},s\right)=\frac{\phi_{i}\left(\text{k},s\right)}{\phi_{n}\left(\text{k},s\right)}, \end{array}$$

(33) $$\begin{array}{l} \;=\frac{V\left(s\right)U\left(s\right)\exp\left(-s\tau_{es}\right)}{O\left(s\right)\left[M\left(s\right)R\left(s\right)-N\left(s\right)P\left(s\right)\exp\left\{-s\left(\tau_{es}+\tau_{se}\right)\right\}\right]}. \end{array}$$

where

(34) $$\begin{array}{l} O\left(s\right)=1-G_{ii}L_{ii}, \end{array}$$

(35) $$\begin{array}{l} V\left(s\right)=G_{ie}L_{ie}\left(s\right)N\left(s\right)+G_{is}L_{is}M\left(s\right). \end{array}$$

#### 2.2.4. Transfer function to TRN from retina

Replacement of ϕ_*s*_ and ϕ_*e*_ in Equation (17) by means of Equations (19) and (31), yields the transfer function to the TRN from retina,

(36) $$\begin{array}{l} T_{rn}\left(\text{k},s\right)=\frac{\phi_{r}\left(\text{k},s\right)}{\phi_{n}\left(\text{k},s\right)}, \end{array}$$

(37) $$\begin{array}{l} =\frac{W\left(s\right)U\left(s\right)}{M\left(s\right)R\left(s\right)-N\left(s\right)P\left(s\right)\exp\left[-s\left(\tau_{es}+\tau_{se}\right)\right],} \end{array}$$

where

(38) $$\begin{array}{l} W\left(s\right)=G_{re}L_{re}N\left(s\right)\exp\left[-s\left(\tau_{es}+\tau_{se}\right)\right]+G_{rs}L_{rs}M\left(s\right). \end{array}$$

#### 2.2.5. Corticothalamic transfer function characteristics

**Figure 2:**
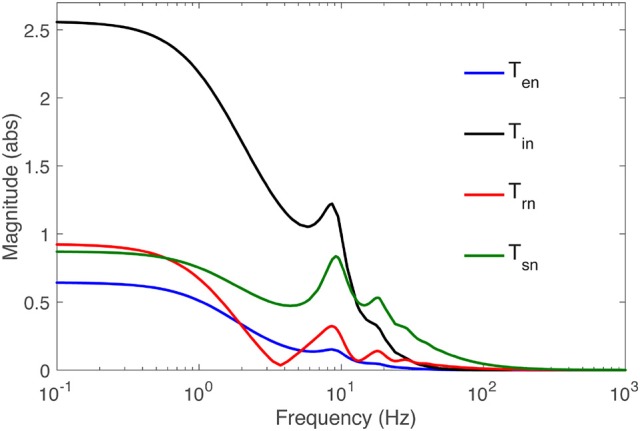
Magnitude of the transfer functions *T*_*an*_ to populations *a* = *s, r, i, e* vs. frequency, as labeled, for **k** = 0 and the parameters in Table 1.

The frequency response of the transfer functions is the transfer function evaluated on the imaginary axis of the *s*-plane, where *s* = −*iΩ*. Figure 2 shows the frequency responses of all populations for the nominal parameter values in Table 2, using the Control System Toolbox of Matlab 2017a to carry out the calculations. More detailed analysis of the model with respect to these parameters can be found in Robinson et al. (2002, 2004) and Abeysuriya et al. (2015). Only the the spatially-uniform effects of perturbations, i.e., **k** = 0, is explored in this study. Low frequencies are passed, while high frequencies are attenuated, and for input signals with small frequency, each transfer function represents an amplifier with constant gain. At higher frequencies, pronounced resonances at 9 and 18 Hz are present in all transfer functions, which can be associated with the alpha and beta peaks in the brain's wake state. The functions become less resonant as the signal gets further away from retina to the cortex: the thalamic functions *T*_*sn*_ and *T*_*rn*_ have higher amplitude and wider bandwidth than the cortical ones *T*_*en*_ and *T*_*in*_.

**Table 2:** Estimated (dimensionless) synaptic gains for normal adults in the alert, eyes-open state (Robinson et al., 2004).

**Gain**	**Value**	**Gain**	**Value**
*G*_*ee*_	6.8	*G*_*ie*_	6.8
*G*_*se*_	2.5	*G*_*re*_	1.0
*G*_*ii*_	8.1	*G*_*ei*_	−8.1
*G*_*sr*_	−1.9	*G*_*rs*_	0.19
*G*_*es*_	1.7	*G*_*is*_	1.7
*G*_*sn*_	0.8	*G*_*sn*_	0.8

The transfer function fully describes the linear system properties and enables us to investigate its response to any external signal. Setting the denominator of the transfer function to zero yields the characteristic equation of the system, whose roots are its eigenvalues and mark the poles; these poles determine the basic modes into which the system response can be decomposed. Furthermore, all corticothalamic transfer functions calculated above, have some or all of their poles (basic modes) in common, which is a direct result of the interconnectedness of the system. Roots of the numerator of the transfer function are the zeros of the system; signals at these frequencies are not transfered through the system.

## 3. Results

### 3.1. Control systems interpretation of corticothalamic transfer functions

In this section, we decompose the transfer functions into elementary modes whose behaviors we associate with data filters whose control system properties are well understood. Only the spatially-uniform effects of perturbations, i.e., he simpler the description of the system, alt **k** = 0, have been explored in this study.

#### 3.1.1. Reduced model

The corticothalamic transfer functions, are ratios of exponential polynomials of *s*. We approximate each transfer function *T*_*ab*_(*s*) by a rational function, whose properties can be interpreted in terms of data filters (Ogata and Yang, 1970; Kwakernaak and Sivan, 1972), with

(39) $$\begin{array}{l} T_{ab}\left(s\right)=\frac{q\left(s\right)}{p\left(s\right)}=\frac{q\left(s\right)}{\overset{n}{\underset{j=1}{\prod }}\left(s+p_{j}\right)}, \end{array}$$

where *q*(*s*) and *p*(*s*) are polynomials of degree *m* and *n*, respectively, with *m* < *n*. Therefore, when the *p*_*j*_ are all distinct (we do not consider degenerate roots here), one has the partial fraction decomposition

(40) $$\begin{array}{l} T_{ab}\left(s\right)=\overset{n}{\underset{j=1}{\sum }}\frac{r_{j}}{s+p_{j}}, \end{array}$$

where the residues *r*_*j*_ are

(41) $$\begin{array}{l} \begin{array}{c} r_{j}=\left(s+p_{j}\right)T_{ab}\left(s\right){|}_{s=-p_{j}}. \end{array} \end{array}$$

The smaller *n* is, the simpler the description of the system, although accuracy is lost if *n* is made too small. In subsequent sections, we seek the smallest *n* that retains the main dynamics. Generally, this leads to the most heavily damped modes (poles with the largest negative real parts) being discarded.

#### 3.1.2. Filter identification

Once we have a few-pole approximation of the system transfer function, we examine it from a control-systems perspective to determine its predictive properties. Using Equation (40), the response ϕ_*b*_ of population *b* to an input signal ϕ_*a*_ can be written as

(42) $$\begin{array}{l} \phi_{b}\left(s\right)=\underset{j}{\sum }\phi_{b}^{j}\left(s\right)=\underset{j}{\sum }\frac{r_{j}}{s+p_{j}}\phi_{a}\left(s\right), \end{array}$$

where Equation (42) defines $\phi_{b}^{j}\left(s\right)$.

A general transfer function will have one or more pairs of complex conjugate poles in the Laplace domain, in addition to one or more real poles. Therefore, each pair of conjugate poles generates a real response mode. We also pair up real poles in the next part of the analysis to conveniently treat both cases together as second order filters whose functions are well known.

Hence, we consider the partial transfer function of the sum of two fractions associated with poles *p*_*j*_ and *p*_*j* + 1_ either both real or conjugate pair, which we denote by $T_{ab}^{J}\left(s\right)$, with

(43) $$\begin{array}{l} T_{ab}^{J}\left(s\right)=K\frac{s+\tau_{p}^{-1}}{\left(s+p_{j}\right)\left(s+p_{j+1}\right)}, \end{array}$$

(44) $$\begin{array}{l} \begin{array}{c} \tau_{p}=\frac{r_{j}+r_{j+1}}{r_{j}p_{j+1}+r_{j+1}p_{j}}, \end{array} \end{array}$$

(45) $$\begin{array}{l} \begin{array}{c} K=r_{j}+r_{j+1}. \end{array} \end{array}$$

Equation (43) yields

(46) $$\begin{array}{l} \phi_{b}^{J}\left(s\right)=\left(s+\tau_{p}^{-1}\right)\left[\frac{K}{\left(s+p_{j}\right)\left(s+p_{j+1}\right)}\right]\phi_{a}\left(s\right), \end{array}$$

(47) $$\begin{array}{l} =H_{ba}^{J}\left(s\right)G_{a}^{J}\left(s\right)\phi_{a}\left(s\right). \end{array}$$

By defining

(48) $$\begin{array}{l} \phi_{a}^{I}\left(s\right)=G_{a}^{J}\left(s\right)\phi_{a}\left(s\right), \end{array}$$

we can write

(49) $$\begin{array}{l} \phi_{b}^{J}\left(s\right)=H_{ba}^{J}\left(s\right)\phi_{a}^{I}\left(s\right), \end{array}$$

(50) $$\begin{array}{l} =H_{ba}^{J}\left(s\right)G_{a}^{J}\left(s\right)\phi_{a}\left(s\right). \end{array}$$

Equations (48) and (49) express how the input ϕ_*a*_(*s*) first passes through $G_{a}^{J}\left(s\right)$ to generate $\phi_{a}^{I}\left(s\right)$, which is transfered via $H_{ba}^{J}\left(s\right)$ to generate the response $\phi_{b}^{J}\left(s\right)$.

The input ϕ_*a*_(*t*) passes through the filter $G_{a}^{J}\left(s\right)$,

(51) $$\begin{array}{l} G_{a}^{J}\left(s\right)=\frac{K}{\left(s+p_{j}\right)\left(s+p_{j+1}\right)}. \end{array}$$

**Figure 3:**
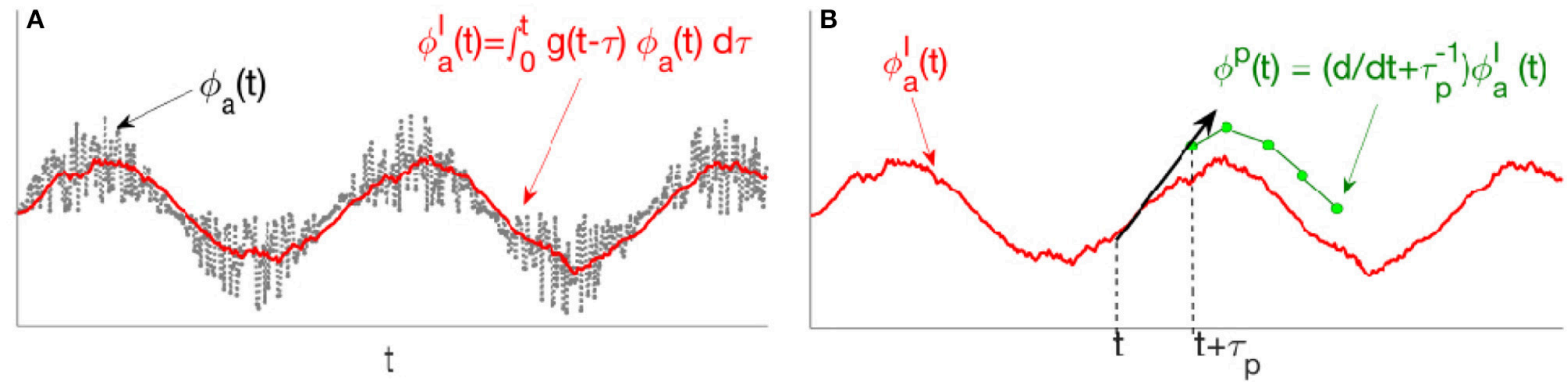
Schematic of stages in the predictor given by Equation (47). **(A)** The input signal ϕ_*a*_(*t*) first passes through $G_{a}^{J}\left(s\right)$, a second-order low-pass convolution filter. **(B)** The filtered signal $\phi_{a}^{I}\left(t\right)$ then passes through $H_{ba}^{J}\left(s\right)$, where, linear extrapolation over a prediction time τ_*p*_ yields the predicted signal ϕ^*P*^(*t*).

Transforming Equation (51) to the time domain yields

(52) $$\begin{array}{l} \phi_{a}^{I}\left(t\right)=\int_{0}^{t}g\left(t-\tau \right)\phi_{a}\left(t\right)d\tau , \end{array}$$

where

(53) $$\begin{array}{l} g\left(t\right)=ke^{-p_{j}t}-ke^{-p_{j+1}t}, \end{array}$$

with

(54) $$\begin{array}{l} k=\frac{K}{\left|p_{j+1}-p_{j}\right|}; \end{array}$$

hence this filter acts as an integrator in the form of a convolution; Figure 3A shows a schematic illustration of this convolution. This filter can be rewritten as a standard second-order filter, as

(55) $$\begin{array}{l} G_{a}^{J}\left(s\right)=\frac{K\Omega_{0}^{2}}{s^{2}+2\zeta \Omega_{0}s+\Omega_{0}^{2}}, \end{array}$$

where *K* is the low frequency gain, Ω_0_ is the natural frequency, ζ is the damping coefficient, and the filter's bandwidth termed as *B* and is 2ζΩ_0_. At *s* ≪ Ω_0_ the right of Equation (55) approaches *K*, while in the opposite limit it approaches *K*/*s*^2^. When ζ > 1, the filter is overdamped and exponentially decays to the steady state without oscillating, while larger values of ζ yield a slower return to equilibrium. A critically damped filter has ζ = 1 and returns to the steady state as quickly as possible without oscillating. When ζ < 1, the filter is underdamped and exhibits damped oscillations. The peak frequency response occurs at ±Ω_*peak*_, with

(56) $$\begin{array}{l} \Omega_{peak}=\{\begin{array}{ll} 0, & \zeta \geq 1/\sqrt{2}\\ \Omega_{0}\sqrt{1-2\zeta^{2}}, & 0\leq \zeta <1/\sqrt{2} \end{array}. \end{array}$$

The peak magnitude of $G_{a}^{J}\left(s\right)$ satisfies

(57) $$\begin{array}{l} G_{a}^{J}\left(i\Omega_{peak}\right)=\{\begin{array}{ll} 1, & \zeta \geq 1/\sqrt{2}\\ \frac{1}{2\zeta \sqrt{1-\zeta^{2}}}, & 0\leq \zeta <1/\sqrt{2} \end{array}. \end{array}$$

**Figure 4:**
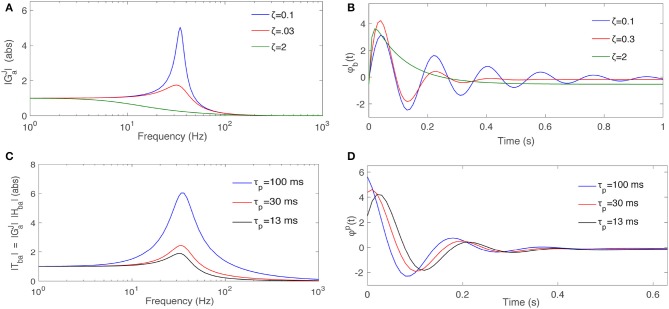
Dependence of filter properties on parameters. **(A)** Magnitude of a typical second order filter $G_{a}^{J}\left(s\right)$ from Equation (55) for Ω_0_ = 35 *s*^−1^, *K* = 1, and various ζ, as labeled. **(B)** Response of $G_{a}^{J}\left(s\right)$ to an impulse input signal for various ζ. **(C)** Magnitude of the total $T_{ba}^{J}=H_{ba}^{J}\left(s\right)G_{a}^{J}\left(s\right)$, for fixed ζ = 0.3 and $H_{ba}^{J}\left(s\right)=\left(s+\tau_{p}^{-1}\right)$ for various τ_*p*_. **(D)** Response of $T_{ba}^{J}$ when the input signal is an impulse function for various τ_*p*_.

Figure 4A shows the behavior of a nominal $G_{a}^{J}\left(i\Omega \right)$ when ζ changes, for fixed Ω_0_ and *K*. Figure 4B shows the response of this filter when the input ϕ_*a*_(*t*) is a Dirac delta function for a the same range of ζ.

The convolved signal $\phi_{a}^{I}$ is transferred through the filter $H_{ba}^{J}\left(s\right)$. By transforming Equation (49) into the time domain one then finds

(58) $$\begin{array}{l} \phi_{b}^{J}(t)=\tau_{p}^{-1}\phi^{P}(t), \end{array}$$

(59) $$\begin{array}{l} \phi^{P}(t)=\tau_{p}\frac{d}{dt}\phi_{a}^{I}(t)+\phi_{a}^{I}(t), \end{array}$$

where τ_*p*_ is termed the prediction time and $\phi_{a}^{P}$ the predicted signal. Hence, the filter $H_{ba}^{J}$ predicts its own input a time τ_*p*_ in the future, which is illustrated in Figure 3B. Figure 4C shows the magnitude of the $T_{ba}^{J}$ for the same nominal $G_{a}^{J}\left(i\Omega \right)$, ζ = 0.3, and $H_{ba}^{J}\left(s\right)=\left(s+\tau_{p}^{-1}\right)$ when τ_*p*_ varies. Figure 4D shows how *T*_*ba*_ predicts the Dirac delta function when τ_*p*_ varies. At larger τ_*p*_ the response signal attains its final steady state value more slowly with a smaller resonant peak and less oscillation.

The above convolution plus prediction processes can be interpreted as a Proportional-Integral-Derivative (PID) control scheme used in engineering control systems (Ogata and Yang, 1970; Kwakernaak and Sivan, 1972). Specifically, in the time domain Equation (46) becomes

(60) $$\begin{array}{l} \phi_{b}^{J}\left(t\right)=\left(\frac{d}{dt}+\frac{1}{\tau_{p}}\right)\int_{0}^{t}g\left(t-\tau \right)\phi_{a}\left(t\right)d\tau , \end{array}$$

which is a continuous-time serial PID. In this equation the integral (I) smooths the input signal to reduce the effects of noise. The smoothed signal is then extrapolated a time τ_*p*_ into the future by the combined effects of the proportional (P) and derivative (D) operators in the first parentheses on the right. Hence, each pair of poles in Equation (40) yields a partial transfer function that can be interpreted as a PID controller.

### 3.2. Control system interpretation of corticothalamic dynamics

We now examine each corticothalamic transfer function from Section 2.2 to determine its control-systems characteristics.

#### 3.2.1. Corticothalamic filters

**Figure 5:**
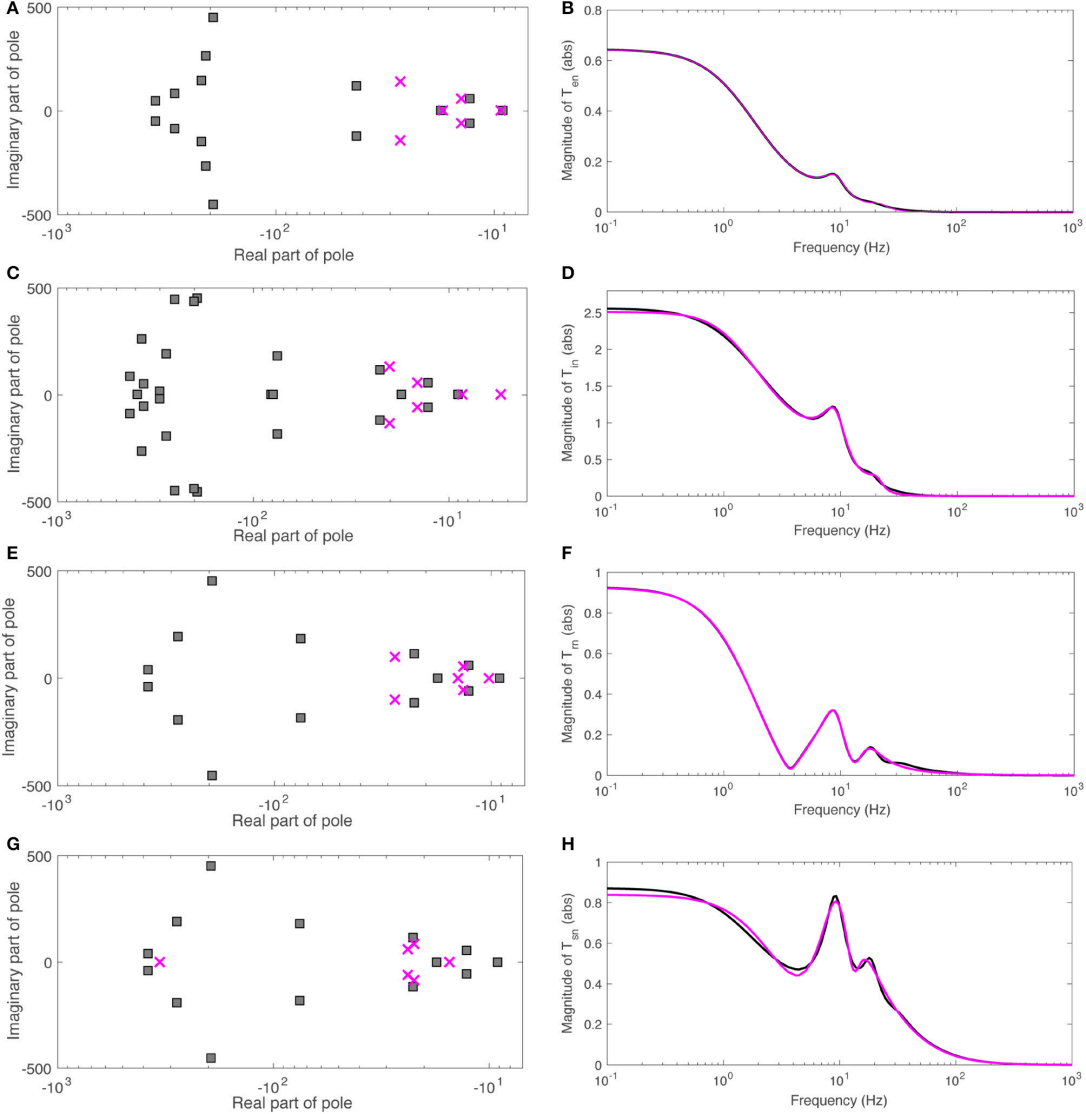
Poles and magnitudes of rational approximations to the transfer functions *T*_*an*_. **(A)** Poles of 16-pole (black squares) and 6-pole (red crosses) approximations of *T*_*en*_. **(B)** Magnitude of 16-pole (black) and 6-pole approximations of *T*_*en*_ vs. frequency. **(C)** Same as **(A)** for *T*_*in*_. **(D)** Same as **(B)** for *T*_*in*_. **(E)** Same as **(A)** for *T*_*rn*_. **(F)** Same as **(B)** for *T*_*rn*_. **(G)** Same as **(A)** for *T*_*sn*_. **(H)** Same as **(B)** for *T*_*sn*_.

We find that a 16-pole approximation of *T*_*en*_ is accurate to within a root-mean-square (rms) fractional error of 0.01 over the frequency range 0 to 150 Hz for the parameters in Table 2, while a 6-pole approximation suffices for most purposes, accurate to within an rms fractional error of 0.02 over the same range. Figure 5A shows the poles of the 16- and 6-pole approximations of *T*_*en*_, while Figure 5B shows the magnitudes of both functions. These results show that the 6-pole approximation retains the main features of the dynamics and is sufficient for analyzing its effects; in most cases, it exhibits slightly shifted versions of the least-damped poles of the 16-pole approximation. Similar observations apply to the other transfer functions *T*_*in*_, *T*_*rn*_, and *T*_*sn*_ whose 6-pole approximations are accurate to within an rms fractional error of 0.02. The pole maps and the frequency responses for these approximations are plotted in Figures 5C–H. We used the Control System Toolbox of Matlab 2017a to carry out these approximations.

Turning to filter analysis, six poles can be summed in pairs that dominate at low (*f* ≲ 5 Hz), alpha (5 Hz ≲ *f* ≲ 15 Hz) and beta (15 Hz ≲ *f*) frequency ranges, respectively. We thus write

(61) $$\begin{array}{l} T_{bn}^{r}\left(s\right)=\overset{6}{\underset{j=1}{\sum }}\frac{r_{j}}{s+p_{j}}, \end{array}$$

(62) $$\begin{array}{l} =T_{bn}^{low}\left(s\right)+T_{bn}^{alpha}\left(s\right)+T_{bn}^{beta}\left(s\right), \end{array}$$

where *b* = *s, r, i, e* and $T_{bn}^{low}$ is the sum of the two real poles while $T_{bn}^{alpha}$ and $T_{bn}^{beta}$ are the sums over the complex conjugate pairs of poles that represent oscillatory responses in the alpha and beta frequency ranges, respectively; in each case *p*_*j*_ = Γ_*j*_ ± *iΩ*_*j*_.

#### 3.2.2. Filter properties of $T_{bn}^{low}$

This filter has two poles on the negative real axis at $p_{1}=\Gamma^{-}$ and $p_{2}=\Gamma^{+}$, with

(63) $$\begin{array}{l} \Gamma^{\pm }=-\left(\zeta \pm \sqrt{\zeta^{2}-1}\right)\Omega_{0}. \end{array}$$

This corresponds to all ζ > 1 and determines that $G_{n}^{low}$ is an overdamped (no oscillation) low-pass filter. The cut-off frequency of $G_{n}^{low}$ is $\Omega_{0}^{low}=\sqrt{\Gamma^{-}\Gamma^{+}}\;{\text{s}}^{-1}$. The parameters of $T_{bn}^{low}$ calculated for populations *b* = *s, r, i, e* are listed in the Table 3. The impulse response takes the form

(64) $$\begin{array}{l} \phi_{bn}^{low}\left(t\right)=r^{-}\exp\left(\Gamma^{-}t\right)+r^{+}\exp\left(\Gamma^{+}t\right). \end{array}$$

**Table 3:** Parameters obtained for low, alpha, and beta filters of corticothalamic transfer functions *T*_*an*_ using filter identification method developed in Section 3.

		***T***_*****en*****_			***T***_*****in*****_			***T***_*****rn*****_			***T***_*****sn*****_			
**Quantity**	**Description**	**Low**	**Alpha**	**Beta**	**Low**	**Alpha**	**Beta**	**Low**	**Alpha**	**beta**	**Low**	**Alpha**	**Beta**	**Unit**
−Γ^−^	damping rate 1^*st*^ pole	9.3	14.1	26.9	8.6	10.2	20	10.2	13.5	27	15.3	23.8	22.3	-
−Γ^+^	damping rate 2^*nd*^ pole	17.2	14.1	26.9	5.5	14.5	20	14.5	13.5	27	337	23.8	22.3	-
Ω_*c*_	cut-off frequency	0	57.4	143	0	56.8	130	0	56.2	101	0	57.9	84	s^−1^
|*K*|	gain	1.8	3.82	1.62	28.7	33.1	3.4	7.44	4.3	8.3	52.2	48.8	4.6	-
ζ	damping coefficient	1.04	0.23	0.18	1.02	0.24	0.15	1.01	0.23	0.26	2.45	0.38	0.25	-
Ω_0_	natural frequency	12.6	59.1	145	6.8	58.6	131	12	57.8	104	71.8	62.6	86.8	s^−1^
*B*	bandwidth	26.5	28.3	53.9	14.2	29	40.2	24.5	27	55.4	352	47.8	44.6	s^−1^
Ω_*peak*_	resonance frequency	0	55.6	140	0	54.9	128	0	54.5	97.2	0	52.7	90	s^−1^
*M*_*peak*_	resonance magnitude	1	2.1	2.7	1	2.1	3.3	1	2.2	2.0	1	1.4	2.0	-
τ_*p*_	prediction time	16	28	6	20	17	2	73	7	15	18	23	2	ms

Because the second exponential decays faster than the first, the response is approximately first-order integrator with a time constant Γ^−^ for large ζ. Thus, poles closest to the positive half of the s-plane dominate the response.

#### 3.2.3. Filter properties of $T_{bn}^{alpha}$ and $T_{bn}^{beta}$

Both filters share the same main features because each comprises a complex conjugate pair of poles. Therefore, we focus on the alpha filter's properties. The poles are complex conjugates lying in the left half of the *s*-plane with *p*_*j*_, *p*_*j*+1_ = Γ ± *iΩ*_*c*_,

(65) $$\begin{array}{l} \Gamma =-\zeta \Omega_{0}, \end{array}$$

(66) $$\begin{array}{l} \Omega_{c}=\Omega_{0}\sqrt{1-\zeta^{2}}, \end{array}$$

where Ω_*c*_ is termed the cut-off frequency and 0 ≤ ζ < 1. The frequency response of $G_{n}^{alpha}$ has a resonance with

(67) $$\begin{array}{l} \Omega_{peak}=\Omega_{0}\sqrt{1-2\zeta^{2}}, \end{array}$$

(68) $$\begin{array}{l} |G_{n}^{alpha}\left(\Omega_{peak}\right)|=\frac{1}{2\zeta \sqrt{1-\zeta^{2}}}, \end{array}$$

as the resonance frequency and magnitude, respectively. Note that Ω_*peak*_ ≠ Ω_*c*_ and that the two frequencies are equal only for ζ = 0, which corresponds to a pure oscillator with Γ = 0, more generally, the resonance bandwidth is 2Γ.

Regarding the temporal response, we must consider the nature of the residues at the poles as well as the pole locations. Since $p_{j}=p_{j+1}^{*}$, these residues are conjugates, so the impulse response of $G_{n}^{alpha}$ is

(69) $$\begin{array}{l} \phi_{n}^{I}\left(t\right)=\frac{\Omega_{0}}{\sqrt{1-\zeta^{2}}}\exp\left(-\Omega_{0}\zeta t\right)\\ \times \cos\left[\left(\Omega_{0}\sqrt{1-\zeta^{2}}\right)t-\frac{\pi }{2}\right]. \end{array}$$

and the predicted signal passed through $H_{bn}^{alpha}$ is

(70) $$\begin{array}{l} \phi_{n}^{alpha}\left(t\right)=\tau_{p}^{-1}\phi_{n}^{p}\left(t\right), \end{array}$$

(71) $$\begin{array}{ll} =2|r|\exp & \left(-\Omega_{0}\zeta t\right)\cos\left[\left(\Omega_{0}\sqrt{1-\zeta^{2}}\right)t-\arg\left(r\right)\right]. \end{array}$$

**Figure 6:**
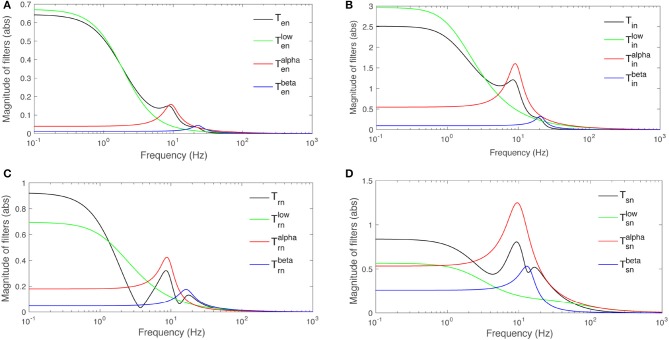
Magnitudes of transfer functions *T*_*an*_ and their low-, alpha-, and beta-frequency parts vs. frequency. Note that the total magnitude is not the sum of the magnitudes of the three parts because of phase difference between them. **(A)** *T*_*en*_. **(B)** *T*_*in*_. **(C)** *T*_*rn*_. **(D)** *T*_*sn*_.

The parameters calculated for this filter are listed in Table 3. The corresponding frequency responses for *b* = *s, r, i, e* are plotted in Figure 6. An alpha rhythm (7.5–12 Hz) is detectable in every population's alpha response, with peak frequences ranging from ≈ 8.4 Hz for the specific nuclei to ≈ 9.3 Hz for inhibitory neurons in the cortex.

Similar results for beta filters are also listed in Table 3 and plotted in Figure 6. The beta responses exhibit resonances in the beta band (12.5−−30 Hz) where amplitudes are significantly smaller than the alpha resonances.

Both alpha and beta filters have higher peaks in thalamus than cortex. Furthermore, in every population except *s*, the damping rate of alpha filters are approximately half of the beta filters', meaning the alpha waves live longer than beta waves in these populations. But, the result shows that alpha and beta waves would last for the same time in the response of population *s* where their damping rates are relatively close. This suggests that beta waves live longer in the LGN response to stimuli than in the rest of the system.

Calculating τ_*p*_ from the filter parameters yields

(72) $$\begin{array}{l} \tau_{p}\Omega_{0}={\left[\sqrt{1-\zeta^{2}}\frac{\text{Im}\left(r\right)}{\text{Re}\left(r\right)}-\zeta \right]}^{-1}, \end{array}$$

and shows that τ_*p*_ is governed by both the filters' poles and their corresponding residues. Alpha and beta filters of the corticothalamic functions have small damping rates, ζ ≪ 1, and therefore the the prediction time in Equation (71) for these filters can be approximated as

(73) $$\begin{array}{l} \tau_{p}\Omega_{0}\approx \frac{\text{Re}\left(r\right)}{\text{Im}\left(r\right)}. \end{array}$$

The quantity τ_*p*_ Ω _0_ represents what portion of its resonance cycle the filter predicts in advance. One significant observation is that alpha and beta filters of populations *e*, *i* and *s* present very similar patterns of having τ_*p*_ of alpha filters longer than those of their beta filters. In contrast, the population *r* has the opposite relation, which means its beta filter predicts further in advance.

### 3.3. Simulation with random input signal

Aside from the issue of how to interpret corticothalamic dynamics in terms of data filters *per se*, there is the central question of how well these filters enable the system to predict its complex input signals out to some horizon in the future.

The corticothalamic system can only respond significantly to signals out to approximately the flicker fusion frequency of around 20 Hz. Hence, as a particularly severe test of its prediction capabilities, we simulate the system response to white noise, bandwidth limited to 30 Hz, with total power *P*_*n*_. We are interested in time-varying, but spatially unstructured stimuli. The perturbation analysis then corresponds to presenting the entire filed of view with a stimulus that consists of a sequence of luminances that fluctuates according to a small amplitude (perturbation) around a base level of intensity (steady-state). Such stimuli are widely employed in visual flicker experiments to probe SSVEPs (Herrmann, 2001; VanRullen and Macdonald, 2012). We used Control System Toolbox of Matlab 2017a to carry out the calculations of time responses for transfer function *T*_*en*_ using the relevant equations and parameters in the Tables 1, 2, and to generate a random seed for ϕ_*n*_ to stimulate the system. Note that the random noise does not affect spectra or other conclusions; it only changes the specific realization of the time series. We estimate the time offset τ_*d*_ between the measured output and the stimulus and denote the shifted signal by ϕ_*e*_(*t*−τ_*d*_). We then define the difference between the stimulus and the output of each filter as

(74) $$\begin{array}{l} \phi^{R}\left(t\right)=\phi_{n}\left(t\right)-K\phi_{e}\left(t-\tau_{d}\right), \end{array}$$

(75) $$\begin{array}{ll} \approx & \phi_{n}\left(t\right)-\left[\begin{array}{c} k_{l}\;k_{a}\;k_{b} \end{array}\right]\left[\begin{array}{c} \phi_{e}^{low}\left(t-\tau_{d}^{l}\right)\\ \phi_{e}^{alpha}\left(t-\tau_{d}^{a}\right)\\ \phi_{e}^{beta}\left(t-\tau_{d}^{b}\right) \end{array}\right], \end{array}$$

which we term the residual signal. For each filter, we find the optimal *K* that minimizes the power of residual signal, termed as *P*^*R*^. The power of the residuals is minimized to reduce the error between the predicted values and the real coming information subject to temporal frequencies that would normally be encountered in the signal part of sensory input. The resulting parameters are listed in the Table 4.

**Table 4:** Optimal parameters computed for *T*_*en*_ filters, when stimulated with white noise of bandwidth 30 Hz and total power *P*_*n*_.

**Optimization Scheme**	***k*_*l*_**	***k*_*a*_**	***k*_*b*_**	**$P^{R}/P_{n}$**	**τ_*d*_(ms)**
Optimize the individual filters	4.4	0	0	0.53	49
	0	11.2	0	0.39	12
	0	0	28	0.33	6
Optimize the total of sum of filters through a single gain, as in Equation (75)	3.6	3.6	3.6	0.46	51
Optimize the total of sum of filters through separate gains, as in Equation (76)	1.9	8.9	28	0.29	50

**Figure 7:**
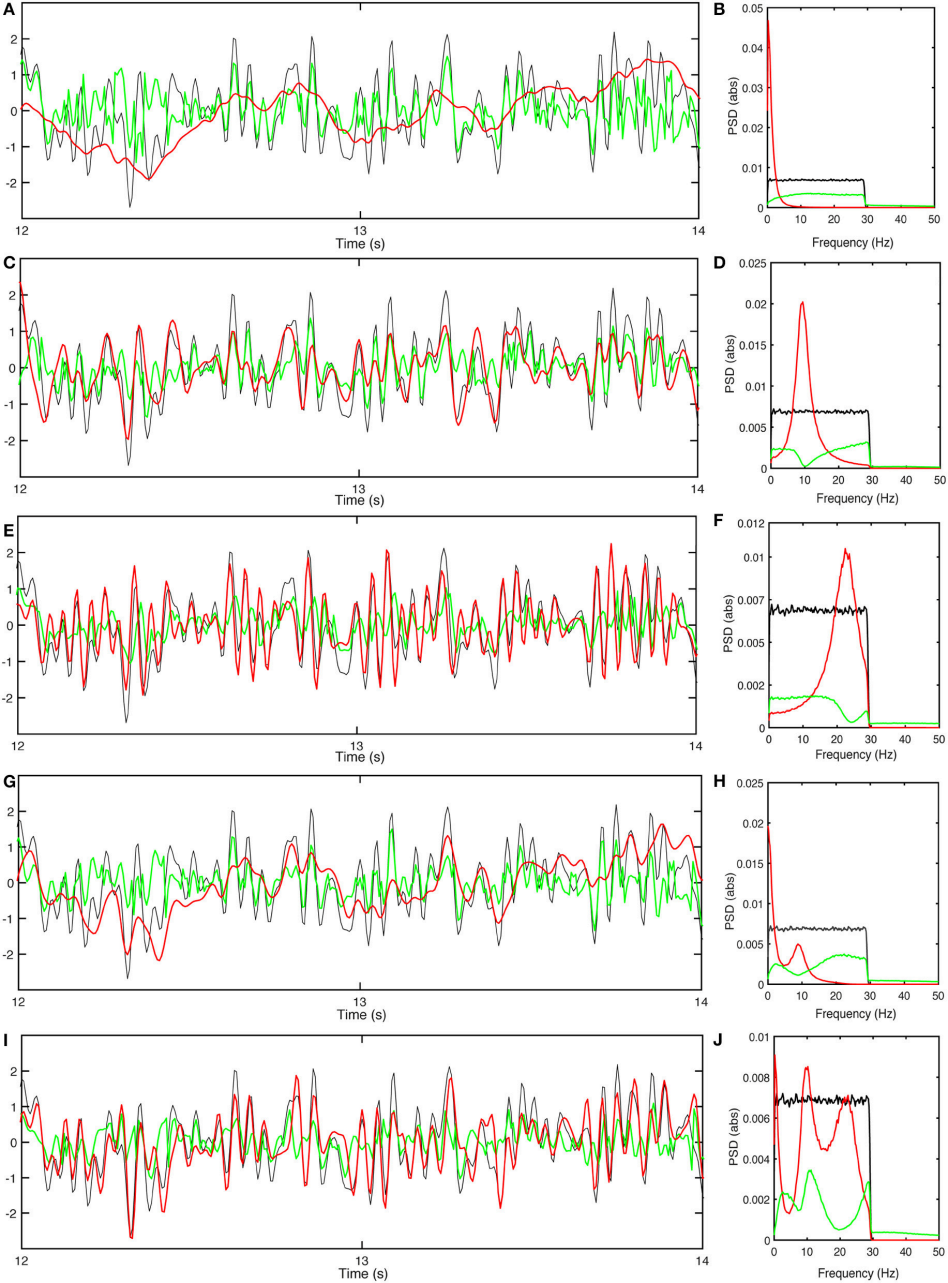
Responses of the population *e* with gain adjustment, and corresponding power spectra. **(A)** Time series of stimulus (black), normalized-shifted filter output *kϕ*_*en*_(*t*−τ_*d*_) (red) and residual signal (green) for $T_{en}^{low}$. **(B)** Power spectra of response signals obtained for $\phi_{en}^{low}$. **(C)** Same as **(A)** for $\phi_{en}^{alpha}$. **(D)** Same as **(B)** for $\phi_{en}^{alpha}$. **(E)** Same as **(A)** for $\phi_{en}^{beta}$. **(F)** Same as **(B)** for $\phi_{en}^{beta}$. **(G)** Same as **(A)** for $\Phi_{en}^{\left(0\right)}$. **(H)** Same as **(B)** for $\phi_{en}^{\left(0\right)}$. **(I)** Same as **(A)** for $\phi_{en}^{\left(\text{I}\right)}$. **(J)** Same as **(B)** for $\phi_{en}^{\left(\text{II}\right)}$.

Figure 7A shows the optimized outputs of the slow filter contribution to *T*_*en*_, and its residual signal compared to the stimuli. This filter predicts the slow trends of the external stimulus, corresponding to its large prediction horizon. Figure 7B shows the power spectra of the corresponding signals, which show that the narrow bandwidth of the filter limits it to predicting slow changes because it suppresses *P*^*R*^ at low frequencies. The filter has a relatively narrow bandwidth which results in an optimal *k*_*l*_ that reduces $P^{R}/P_{n}$ to 0.53.

Figure 7C shows the optimally normalized outputs of the alpha filter contribution to *T*_*en*_, and its residual signal compared to the stimuli. The alpha filter requires larger gain *k*_*a*_ than the low frequency filter and has a short delay time. Figure 7D shows the power spectra of the corresponding signals which show that this filter acts as an alpha band predictor with $P^{R}/P_{n}=0.39$ that cannot forecast higher frequencies or slow trends. Similar results for the beta filter are plotted in Figures 7E,F. The results show a need for a significantly higher gain *k*_*b*_ to achieve the closest fit to the stimuli; however, this filter can reduce $P^{R}/P_{n}$–0.33 because of its significant tail at lower frequencies.

Slow, alpha, and beta filters detect and predict different frequency bands and generate responses, each of which focuses on information in the relevant band. This explains the need for a parallel set of filters normalized so that information about slow trends, and alpha and beta-band oscillations can be retrieved; the best response is obtained by summing the filters. We thus sum the filters in forms in which the gains can be controlled so that the full response is adjusted to optimally track the stimuli. In the first case, we normalize the total sum of the filters through a single factor, *k*, witch might correspond to an overall neuromodulation, for example. This yields

(76) $$\begin{array}{ll} \phi_{en}^{\left(\text{I}\right)} & \approx k\left(\phi_{bn}^{low}+\phi_{bn}^{alpha}+\phi_{bn}^{beta}\right), \end{array}$$

and we adjust *k* so that *P*^*R*^ is minimized. The parameters listed in Table 4 then result in a minimum of $P^{R}/P_{n}=0.46$. Figure 7G plots the normalized-shifted $\phi_{en}^{\left(\text{I}\right)}$ and the residual signal compared to the stimuli for these parameters. Figure 7H shows the corresponding power spectrum plots. The results are quite close to the results of individual low-frequency filter in terms of residual power and time delay, because $T_{en}^{low}$ has the highest magnitude, and normalizing the sum of the filters by a single gain causes it to retain its dominance over the total response.

More accurate tracking of the stimuli could be achieved by tuning the parameters separately for each filter. If the gains *k*_*m*_ could be separately adjusted for *m* = *l, a, b*, denoting the low-frequency, alpha, and beta filters, respectively, then

(77) $$\begin{array}{ll} \phi_{en}^{\left(\text{II}\right)} & \approx k_{l}\phi_{bn}^{low}+k_{a}\phi_{bn}^{alpha}+k_{b}\phi_{bn}^{beta}. \end{array}$$

Such an outcome might well be achievable by the real brain because we know that the strengths of the slow, alpha and beta peaks do not vary in lockstep. Figure 7I shows the response $\phi_{en}^{\left(\text{II}\right)}$ and the residual signal compared to the stimuli for the parameters listed in Table 4, yielding $P^{R}/P_{n}=29$. Figure 7J confirms that this response contains information about slow trends of the stimulus, while alpha and beta band waves are predicted with more emphasis than in $\phi_{en}^{\left(\text{I}\right)}$.

Overall, these results show that the response of population *e* to the stimulus can be approximated by the sum of predicted values. To improve the performance, various normalization constants can potentially be allocated to different filters through gain adjustments. Although we focus on *T*_*en*_ here, the same approach can be used to reveal the mechanisms behind other populations' dynamics and prediction capabilities.

## 4. Discussion

Motivated by the need for a formulation of brain's global dynamics that starts from its physical characteristics (rather than a predetermined formalism or endpoint) and is analytically, numerically, and experimentally tractable, we have used a neural field corticothalamic model to evaluate the transfer functions for various population of neurons. Particular attention has been paid to the understanding of the transfer functions from a control systems perspective. The main results are:

(i) NFT transfer functions for each corticothalamic population were derived and approximated as rational polynomials. These were explored to determine the linear response of each population, and its rate of change, to any stimulus, which are exactly the types of quantities that are used in control systems to enable prediction of future states, and adjustment of gains which is argued to be the analog of implementing attention.

(ii) We approximated transfer functions, while preserving accuracy over the dominant frequency range. These were then used to uncover basic modes by which each population in the corticothalamic system responds to external signals. All corticothalamic transfer functions were then shown to be dominantly governed by a few basic modes and to have similar frequency responses, with different gains.

(iii) Notably, it was shown that each pair of basic modes yields a sub-response that can be expressed in terms of a standard second order PID filter with a low-frequency, alpha, or beta resonance.

(iv) Slow, alpha, and beta filters operate in parallel, with each filter capturing and processing part of the information coming from the external world. The total response to stimuli is obtained by summing these independent responses. This corresponds to the common practice in control systems of using a set of controllers to improve the performance of a tracking system.

(v) Using a random white noise covering the bandwidth of human vision as the stimulus, the responses of corticothalamic filters were simulated. We explored the tracking performance of each filter as well as their sum for the excitatory population in the cortex. The results showed each filter successfully tracks part of the stimulus, while a better prediction is obtained by summing them after separate optimal gain adjustments. This showed how the filters' gains can be adjusted to improve prediction of the future input signal based on its recent time course (value and derives, implicitly) and internal corticothalamic dynamics. Attention, in the other words, may be implemented in the brain through control and adjustment of input gains. This suggested directions for modeling attention in our framework by using mismatches between internal models and external stimuli to drive gain changes. This study will lead to modeling decision and control, and generalization to encompass more general stimuli and sensory systems.

Overall, the results show and interpret the utility of control theory schemes in understanding brain's dynamics, yielding insights into dynamic processes that underlie cognition and action without imposing an outcome in advance.

## Author contributions

TB and PR conceived of the presented idea. TB developed the theory and performed the computations. TB and PR verified the analytical methods. TB worked out the technical details, performed numerical calculations, produced figures and numerical results. TB and PR discussed all the results. TB drafted the first manuscript and both authors contributed to the final manuscript.

### Conflict of interest statement

The authors declare that the research was conducted in the absence of any commercial or financial relationships that could be construed as a potential conflict of interest.
